# Potential APOBEC-mediated RNA editing of the genomes of SARS-CoV-2 and other coronaviruses and its impact on their longer term evolution

**DOI:** 10.1016/j.virol.2020.12.018

**Published:** 2021-04

**Authors:** Jeremy Ratcliff, Peter Simmonds

**Affiliations:** Nuffield Department of Medicine, Peter Medawar Building for Pathogen Research, University of Oxford, Oxford, OX13SY, UK

**Keywords:** SARS-CoV-2, Coronavirus, Virus evolution, APOBEC, Innate immunity

## Abstract

Members of the APOBEC family of cytidine deaminases show antiviral activities in mammalian cells through lethal editing in the genomes of small DNA viruses, herpesviruses and retroviruses, and potentially those of RNA viruses such as coronaviruses. Consistent with the latter, APOBEC-like directional C→U transitions of genomic plus-strand RNA are greatly overrepresented in SARS-CoV-2 genome sequences of variants emerging during the COVID-19 pandemic. A C→U mutational process may leave evolutionary imprints on coronavirus genomes, including extensive homoplasy from editing and reversion at targeted sites and the occurrence of driven amino acid sequence changes in viral proteins. If sustained over longer periods, this process may account for the previously reported marked global depletion of C and excess of U bases in human seasonal coronavirus genomes. This review synthesizes the current knowledge on APOBEC evolution and function and the evidence of their role in APOBEC-mediated genome editing of SARS-CoV-2 and other coronaviruses.

## Highlights

- SARS-CoV-2 sequence variants contain an overabundance of C- > U transitions
- C- > U transitions are the hallmark of the activity of APOBEC cytosine deaminases
- Further work is needed to determine APOBEC's role in coronavirus evolution

## 1 Introduction

The sustained spread of SARS coronavirus 2 (SARS-CoV-2), coupled with readily deployable and accessible sequencing technologies, has given researchers an unprecedented ability to track virus evolution from spillover through early adaptation to human hosts. Mining this data, several authors have identified an overabundance of homoplastic cytidine to uridine transitions in the SARS-CoV-2 genome, particularly in the context of 5′-UC-3′, resulting in a large proportion of nonsynonymous mutations. This mutational pattern is similar to that induced by the cytidine deaminases of the apolipoprotein B mRNA editing enzyme, catalytic polypeptide-like (APOBEC) family of proteins. The ability of this enzyme family to modify retroviruses, retroelements, and some DNA viruses is well established, while their potential to modify RNA viruses is exploratory. This review will provide an overview of the current knowledge of APOBEC function and evolution, the evidence for this mutational phenomenon in coronaviruses, and its potential evolutionary consequences for RNA viruses.

## 2 Discovery of the APOBEC family

In 1987, two groups identified that tissue-specific expression of ApoB100 and ApoB48 from the ApoB gene depended on post-transcriptional modifications of messenger RNA rather than differences in their genomic content (Chen et al., 1987; Powell et al., 1987). In both human and rat enterocytes, a cytosine to uracil modification (C6666U) generated a UAA stop codon and translation of a truncated version of ApoB48 with a modified biological function. In 1993, the protein catalyzing this reaction was isolated from rat enterocytes and designated apo B mRNA editing protein (REPR) (Teng et al., 1993). Shortly thereafter, the existence of other mRNA editing enzymes functioning through a similar catalytic subunit was hypothesized, and the enzyme responsible for ApoB48 was renamed *Apolipoprotein B mRNA Editing Catalytic Polypeptide 1* (APOBEC1) (Davidson et al., 1995). Since that time, many APOBEC1-related genes and splice variants have been identified in the genomes and transcriptomes of fish, mammals, non-human primates, and humans, which collectively are referred to as the APOBEC family of proteins. In humans, the family of cytidine deaminases has 11 members: APOBEC1 (A1), APOBEC2 (A2), seven APOBEC3s (A3A, A3B, A3C, A3D, A3F, A3G, A3H), APOBEC4 (A4), and activation induced deaminase (AID) (Jarmuz et al., 2002; Rogozin et al., 2005). The proteins possess a shared, highly conserved zinc-dependent deaminase domain (ZDD) but are otherwise diverse in function, having been implicated in a plethora of diverse biological pathways including lipid metabolism, antibody diversification, restriction of viruses and transposable elements, and cancer (Burns et al., 2015; Green et al., 2016; Henderson and Fenton, 2015; Roberts et al., 2013; Smith, 2017; Taylor et al., 2013).

## 3 Conservation of structure

Members of the APOBEC family of proteins are readily recognized through their possession of a highly conserved ZDD motif His-X-Glu-X_23-28_-Pro-Cys-X_2-4_-Cys that is present in the cytidine deaminase fold (CD) (Salter et al., 2016). In those maintaining enzymatic activity, the active site of the ZDD coordinates a zinc ion that activates a water for nucleophilic attack of C4 of the pyrimidine ring of C, which in conjunction with the glutamic acid residue leads to the deamination of the base to U. The CD fold is nested in a conserved super-secondary structure of a five-stranded β-sheet surrounded by six α-helices, with minor variations in the X residues potentially leading to the diverse functions and targets of this family of proteins (Salter et al., 2016). A3B, A3F, and A3G contain double deaminase domains where the N-terminal domain is no longer catalytically active for deamination of ssDNA (Salter et al., 2016). In humans, all members of the family have multiple exons and some have been shown to produce splice variants (Harari et al., 2009; Lee et al., 1998; van Maldegem et al., 2009).

## 4 Diversity of function in humans

A1 primarily functions to increase diversity in host-expressed RNAs in a tissue-specific manner (Smith, 2017). The RNA binding and catalytic activity of A1 is dependent on the binding of one of two cofactors - A1 complementation factor (ACF) or RBM47 (Fossat et al., 2014; Mehta et al., 2000) – and, in addition to ApoB, edits NF1, eIF4G, and the 3′UTRs of other host mRNAs (Rosenberg et al., 2011; Skuse et al., 1996; Yamanaka et al., 1997). ACF binds an 11-nt ‘mooring sequence’ 3′ of the target cytidine residue that partially, but not completely, determines the specificity of the nucleotide target (Backus and Smith, 1991; Blanc et al., 2014; Rosenberg et al., 2011). A1 deaminates cytidines in the context of 5′-AC-3′ with the mooring sequence 4–6 nt downstream of the target and is primarily found in the cytoplasm but can traffic to the nucleus. However, A1 is the exception and not the rule to this family, as most APOBEC proteins are regarded for their ability to deaminate C to dU in ssDNA rather than RNA.

AID and AID-like enzymes are an essential component of the adaptative immune system in all jawed vertebrate species (Flajnik and Kasahara, 2010; Rogozin et al., 2007). In humans, AID drives somatic hypermutation and class-switch recombination in B cells through recruitment of DNA repair enzymes following introduction of dU into immunoglobulin V and C genes (Chaudhuri et al., 2003; Dickerson et al., 2003; Muramatsu et al., 1999, 2000; Pham et al., 2003). The introduced dU can lead to transitions upon DNA replication or stochastic introduction of insertions or deletions during mismatch repair, leading to the diversity that is needed to expand the antibody repertoire of the host. AID preferentially deaminates in the 5′-WRC-3′ context (W = A or T, R = A or G) and is basally present in the cytoplasm but traffics to the nucleus to perform immunoglobulin gene editing.

A2 has roles in skeletal and cardiac muscle differentiation (Etard et al., 2010; Liao et al., 1999; Sato et al., 2010; Vonica et al., 2011). Transcripts of eukaryotic translation initiation factor 4 gamma 2 (Eif4g2) and phosphatase and tensin homolog (PTEN) have been suggested as mRNA targets in A2-overexpressing hepatocytes (Okuyama et al., 2012). However, transgene expression of A1 in hepatocytes also leads to Eif4g2 editing (Yamanaka et al., 1995, 1997), so it is unclear if this represents the *in vivo* function or is an artifact of the overexpression model. A4 was discovered in humans through bioinformatic identification and contains orthologs in other mammals, chickens, and frogs (Rogozin et al., 2005). The function of the protein is as of yet unknown but expression is upregulated in the testes of mice, leading the authors to suggest a potential role in spermatogenesis. An A4 homolog was recently cloned from chicken cells and showed *in vitro* antiviral activity against Newcastle disease virus, even though editing of the viral genome was not described (Shi et al., 2020). Neither A2 nor A4 have described target sequences. A2 shows a distributed intracellular distribution while the cellular localization of A4 remains poorly characterised.

The principal activity of the A3 subfamily of proteins is the restriction of viruses and mobile genomic elements, which is covered extensively below. A3A, A3B, A3C, A3D, A3F, and A3H target sequences with a 5′-TC-3′ motif, while A3G targets 5′-CC-3’. Recently, McDaniel et al. investigated the relevance of sequence context adjacent to a TC motif and secondary structure elements to A3 activity on DNA oligonucleotides (McDaniel et al., 2020). Flanking sequence preference varied between subfamily members and within difference structural contexts. In general, A3A, A3F, and A3H preferred A or T flanking the target motif and all members of the subfamily preferred the edited site to be presented outside of defined secondary structure elements. A3A, A3C, and A3H show a broadly distributed intracellular distribution, while A3B is predominantly nuclear, and A3D, A3F, and A3G are primarily localized in the cytoplasm.

Intriguingly, both A3A and A3G have recently been experimentally shown to edit host mRNAs, with A3A-driven editing in activated innate immune cells and A3G-driven editing in hypoxic natural killer and CD8^+^ T cells and overexpressing HEK293T cells (Sharma et al., 2015, 2016, 2019). As all A3s have demonstrated RNA binding activity (Salter et al., 2016), it does open up the intriguing possibility of deamination of RNA in specific cellular contexts by members of the A3 subfamily. Perhaps reflecting the structure dependence of A3-meditated editing of DNA oligonucleotides (McDaniel et al., 2020), it had been shown that editing of host mRNA in activated macrophages and monocytes by A3A occurred preferentially at sites flanked by short palindromic sequences (Sharma et al., 2015). Using bioinformatic approaches, the authors further demonstrated that 72% of edited sites in monocytes and 67% of edited sites in macrophages were present in the loop of a predicted stem-loop structure. In both cell lines, the most common flanking sequence was an upstream A and a downstream A/G, consistent with context preferences for ssDNA targets (McDaniel et al., 2020). C was also commonly seen in the −3 position.

### 4.1 Deaminase-dependent restriction of viruses and mobile genetic elements

**Human Immunodeficiency Virus (HIV-1).** The inhibition of HIV-1 replication through the function of A3D, A3F, A3G, and A3H is the most extensively studied of the viral restriction phenotype of the APOBEC family and has been well reviewed elsewhere (Aydin et al., 2014; Delviks-Frankenberry et al., 2020; Desimmie et al., 2014; Haché et al., 2006; Harris and Dudley, 2015; Xu et al., 2020). During production of new virions in a productively infected host cell, A3 proteins form ribonucleoprotein complexes with HIV-1 RNA and the nucleocapsid domain of the Gag protein. The A3 proteins are subsequently packaged and encapsidated in the mature HIV-1 virions. The major hypermutation events take place during subsequent cellular infection. After viral entry and uncoating, A3 proteins co-localize with HIV-1 RNA during reverse transcription of nascent negative-strand viral cDNA. This single-stranded cDNA acts as the template for plus-strand genomic DNA synthesis and is highly susceptible to the cytidine deamination activities of the packaged A3 proteins. The C→U transitions in the template strand (negative-sense strand) result in G→A transitions in the genomic strand (positive-sense strand) (Mangeat et al., 2003). Many hypermutated proviruses are degraded during or after generation of the cDNA template strand, potentially through a pathway mediated by uracil DNA glycosylase-2, and do not integrate in the host cell genome (Kaiser and Emerman, 2006; Langlois and Neuberger, 2008; Weil et al., 2013; Yang et al., 2007). A large portion of those proviruses that do successfully integrate are rendered defective due to an abundance of mutated start codons and nonsense mutations in several open reading frames (Bruner et al., 2016; Janini et al., 2001; Kieffer et al., 2005).

HIV-1 and other retroviruses contain a potent counteractor of APOBEC restriction – the accessory protein viral infectivity factor (Vif). The early 2000s contained a cascade of information regarding the interaction between Vif and A3G (Conticello et al., 2003; Kao et al., 2003; Marin et al., 2003; Mehle et al., 2004a, 2004b; Sheehy et al., 2002, 2003; Stopak et al., 2003; Yu et al., 2003). Per the current model, Vif recruits an E3 ubiquitin ligase complex composed of Elongin B/C, cullin 5, RING-box subunit 2 (Rbx2), and core-binding factor (CBFβ) to polyubiquitinate A3G, leading to degradation via the host proteasome (Fribourgh et al., 2014; Guo et al., 2014; Jäger et al., 2011; Zhang et al., 2011). Vif-like proteins have been described for other lentiviruses including simian immunodeficiency virus, feline immunodeficiency virus, bovine immunodeficiency virus, jembrana disease virus, visna/maedi virus, and caprine arthritis encephalitis virus (Kristbjörnsdóttir et al., 2004; Nakano et al., 2017; Wang et al., 2018; Zhang et al., 2014; Zhao et al., 2019; Zielonka et al., 2010). Species-specific spumaretrovirinae, another subfamily of retroviruses, infect a diverse number of mammals and encode the accessory protein Bet, which impairs the activity of A3G through direct binding and sequestration rather than targeted degradation (Jaguva Vasudevan et al., 2013; Löchelt et al., 2005; Russell et al., 2005). The presence of APOBEC-antagonism by protein-protein interactions in lentiviruses that infect diverse placental mammals strongly suggests active antiviral APOBEC activity in these representative species. As an alternative mechanism, human T-Cell leukemia virus (HTLV-1) avoids APOBEC activity simply through exclusion of A3G from the packaged virus (Derse et al., 2007). The diverse approaches of retroviruses to avoid A3 activity highlight the importance of this subfamily for restricting this class of viruses.

**Retrotransposons and Mobile Genetic Elements.** Transposable elements (TEs) are selfish genetic elements that are able to move within the genome via one of two methods (Boeke et al., 1985; Bourque et al., 2018; Rubin et al., 1982). Class I TEs (retrotransposons) replicate through a “copy and paste” mechanism wherein reverse transcription of an RNA intermediate produces a cDNA copy of the genetic element that integrates elsewhere in the genome. Class II TEs (DNA transposons) utilize a “cut and paste” mechanism via the activity of an encoded transposase enzyme and do not require RNA intermediaries. The evolution of the human genome has been shaped by mobile genetic elements, and estimates suggest that 46% of it may be comprised of inactivated TEs (Biémont and Vieira, 2006; Lander et al., 2001). In humans, only a subset of long-interspersed element-1 (LINE-1) retrotransposons and short interspersed nuclear elements (SINEs) remain retrotransposition-competent (Brouha et al., 2003; Richardson et al., 2015; Schumann, 2007). The majority of APOBEC family activity on retroelements has been demonstrated on LINE-1 elements *in vitro*. A3A inhibits LINE-1 retrotransposition through a deaminase-dependent mechanism (Richardson et al., 2014) while AID, A1, A3B, A3C, and A3F act through a deaminase-independent pathway (Ikeda et al., 2011; MacDuff et al., 2009; Pizarro and Cristofari, 2016). It has been hypothesized that the massive reduction in active LINE-1 elements in the primate taxon compared to rodentia could be the result of extensive duplication and expansion of the A3 gene (Ostertag and Kazazian, 2001; Schumann, 2007). However, analysis of the depletion of A3 targeted sequence motifs in human retroelements only demonstrated the footprint of A3 activity on a subset of endogenous retroviruses and not in LINE-1 or SINE elements (Anwar et al., 2013).

**DNA viruses.** Beyond retroviruses and TEs, APOBEC-driven C→U/G→A mutations have been demonstrated in a diverse set of ssDNA and dsDNA viruses (Moris et al., 2014). A3B, A3C, A3F, and A3G have been demonstrated to deaminate both the ssDNA negative-sense and positive-sense strand of the pararetrovirus Hepatitis B Virus (HBV) *in vitro* and *in vivo* resulting in a low proportion of G→A hypermutated genomes (Suspène et al., 2005). *In vitro* work has also demonstrated that AID and A1 can edit HBV DNA and HBV DNA and RNA, respectively (Gonzalez et al., 2009; Liang et al., 2013). Murine A1 (mA1) is canonically expressed in hepatic cells and, in a mouse model of HBV infection, was shown to edit transgenic HBV genomes (Renard et al., 2010). The low endogenous expression of AID and A1 in human cells canonically infected with HBV limits the interpretability of their contribution to hypermutation, although AID been shown to be upregulated in human hepatocytes upon infection with hepatitis C virus *in vitro* (Endo et al., 2007; Seeger and Mason, 2000; Uhlén et al., 2015). Human papillomaviruses (HPV), a family of dsDNA viruses, have also been shown to be edited by A3A, A3C, and A3H in A3-transfected 293T cells (Vartanian et al., 2008), while overexpressed A3A, but not A3B or A3C, was able to restrict HPV replication in immortalized keratinocytes (Warren et al., 2015b). Bioinformatic analysis of the HPV genome suggests that the preferred target of A3 enzymes (5′-TC-3′) is significantly depleted, consistent with long-term adaptation to infection of A3 expressing tissues (Warren et al., 2015a). Other DNA viruses shown to be hypermutated by A3 proteins included transfusion-transmitted virus, herpes simplex virus 1, and Epstein-Barr virus (Suspène et al., 2011; Tsuge et al., 2010).

**RNA Viruses.** Although the first description of APOBEC editing activity was on the cellular ApoB mRNA template, direct evidence of APOBEC-driven deamination in RNA viruses has been problematic to demonstrate. The earliest evidence came in 2005 when the rat A1 homolog was shown to edit HIV-1 RNA *in vitro* (Bishop et al., 2004). Recently, it was shown that the restriction of HCoV-NL63, a human-infective alphacoronavirus, by A3C, A3F, and A3H was deaminase-dependent, but, in this case, the authors were unable to demonstrate that this resulted from virus genome editing even after serial passaging of HCoV-NL63 in A3 overexpressing cells (Milewska et al., 2018). Furthermore, while bioinformatic analysis of a large dataset of human-infective viruses demonstrated significant depletion of A3 target motifs (5′-TC-3′) in the DNA viruses parvoviruses, herpesviruses, and papillomaviruses, there was little evidence for this in RNA virus genomes with the exception of the seasonal coronaviruses HCoV-NL63, HCoV-OC43, HCoV-HKU1, and HCoV-229E (although oddly this signature was not detected in the recently emerged MERS-CoV, SARS-CoV-1 and SARS-CoV-2 or their zoonotic source viruses) (Poulain et al., 2020). However, analysis of immunodeficiency-related vaccine-derived rubella virus in the granulomas of infected children did demonstrate a preponderance of C→U transitions and depletion of 5′-TC-3′, accounting for 30% of the transitions when compared to the virus strain used in vaccine manufacture (Perelygina et al., 2019).

**Deaminase-Independent Restriction and Insensitivity.** Deaminase-independent restriction has been demonstrated primarily using overexpression models of catalytically inactive A3 mutants. This phenomenon has been reported for many viruses, including HIV-1, measles, mumps, respiratory syncytial virus, HCoV-NL63, adeno-associated virus, enterovirus A71, and minute virus of mice (Chen et al., 2006; Fehrholz et al., 2012; Li et al., 2018; Narvaiza et al., 2009). Long-interspersed element 1 is also inhibited by A3B and A3F in a deaminase-independent manner (Stenglein and Harris, 2006). In HIV-1, the prevailing model for this restriction is binding of genomic RNA inhibiting the initiation of reverse transcription, which may also explain the phenotype in LINE-1. The mechanism in other RNA and DNA viruses is not well described. Influenza virus and vaccinia virus are not restricted by A3 overexpression and appear to avoid targeting through unknown mechanisms (Kremer et al., 2006; Pauli et al., 2009).

### 4.2 APOBEC origins, diversification, and evolution

**Fig. 1:**
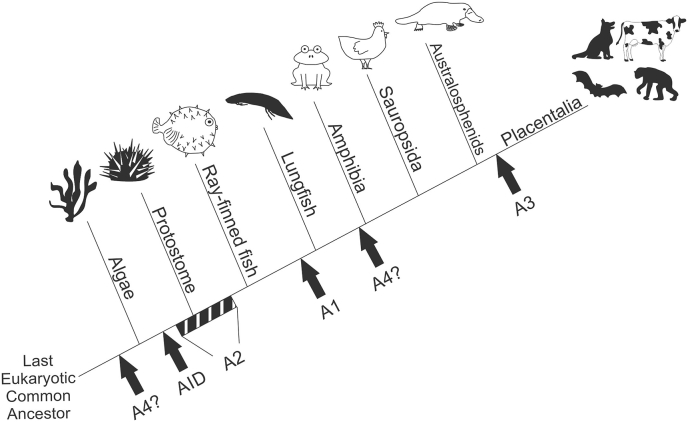
Emergence of APOBEC proteins. Branch length is not drawn to timescale. Question marks for A4 protein represent a lack of concrete evidence at the divergence of amphibians (Rogozin et al., 2005) and putative evidence in algae species (Krishnan et al., 2018). Block for A2 represents uncertainty on emergence prior to protostome - deuterostome split. A1 and A3 both arose from duplication of AID.

**Origin.** The APOBEC family is related to bacterial, yeast, and plant deaminases all possessing highly conserved amino acid motifs responsible for coordination of zinc in the active site (Conticello et al., 2005; Gerber and Keller, 2001; Iyer et al., 2011) (Fig. 1). Bioinformatic analysis suggests that A4 may be the ancient member of the APOBEC family, as homologs have been identified in the phylum cnidaria and occasionally in algal lineages (Krishnan et al., 2018). Another recent study identified, cloned, and demonstrated deaminase activity in AID homologs from sea urchins and branchiopods (Liu et al., 2018). The AID homologs showed increased expression in barrier tissues and were upregulated in response to bacterial challenge, suggesting a potential role in antimicrobial defense. Ancestral members of the family at the point of vertebrate speciation include both AID and A2, and homologs of the two proteins have been identified bioinformatically in birds, amphibians, and ray-finned fish (zebrafish and pufferfish), with an AID homolog responsible for diversity in variable lymphocyte receptors of jawless vertebrates (Conticello et al., 2005; Hsu, 2016). A1 and A3 both arose from independent duplication events of AID (Conticello et al., 2005). A1 has been identified in birds, reptiles, amphibians, and lungfish, suggesting it arose prior to the tetrapod-lungfish divergence (Krishnan et al., 2018). A3 emerged from AID duplication in placental mammals and has undergone frequent duplication, fusion, and loss in different branches of the clade (Krishnan et al., 2018; Munk et al., 2012).

**Fig. 2:**
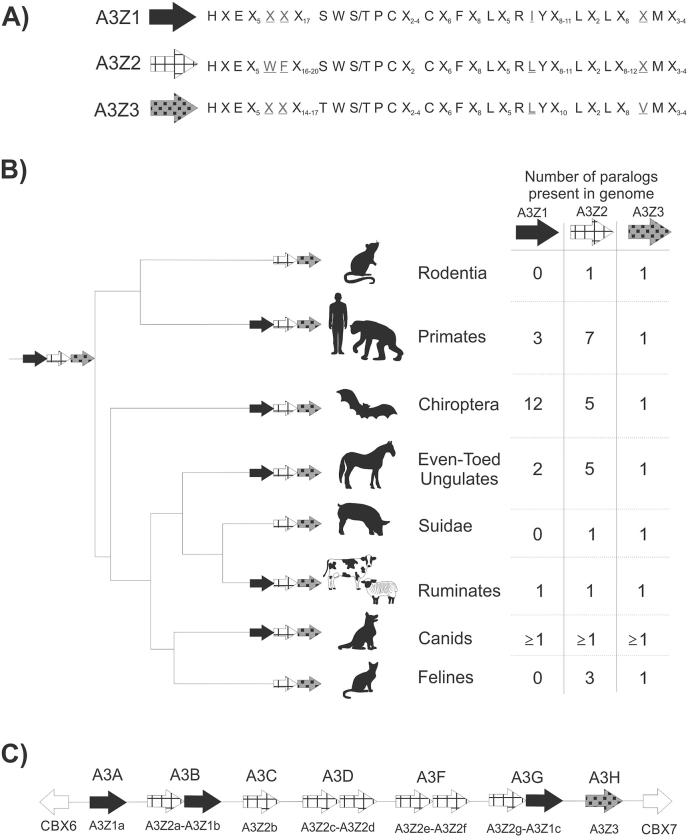
Expansion of A3 paralogs. **(A)** Amino acid motifs within the zinc deaminase domain of the three A3 paralogs. Differences highlighted in grey and underlined. **(B)** Expansion of paralogs in different branches of placental mammals. A3Z1 has been independently lost in several taxonomic classes. Branch length is not drawn to timescale. Table lists number of paralogs and not number of gene products. Taxonomic relationships designed in consultation with the Tree of Life web project. **(C)** Diagram of the A3 gene locus in humans and primates. Adjacent paralogs (e.g. A3Z2a-A3Z1b) represent fused paralogs in a single gene product.

**A3 paralog expansion.** A3 homologs are not present in marsupials or monotreme genomes and appear to be exclusive to placental mammals (Ito et al., 2020; Mikkelsen et al., 2007; Warren et al., 2008). The A3 proteins contains three paralogs (A3Z1, A3Z2, A3Z3) that are designated according to conservation of amino acid residues within the zinc-coordinating domain (LaRue et al., 2009) (Fig. 2a). From the most recent common ancestor of the three A3 paralogs, A3Z3 may be the most ancient having diverged approximately 250 million years ago with A3Z1 and A3Z2 diverging from each other 50 million years later (Munk et al., 2012). It is hypothesized that the most recent common ancestor of placental mammals contained a gene set of A3Z1-A3Z2-A3Z3 that, through multiple processes of differential evolution, have led to the diversity seen in extant species (Fig. 2b). Mapping and identification of A3 genes takes advantage the presence of genes CBX6 and CBX7 upstream and downstream of the gene locus, respectively. For the purpose of this article, particular attention will be played to species known to be hosts for coronaviruses – bats and humans – but we draw attention to an extensive review of the activity and characteristics of feline A3s (Zhang et al., 2018).

The complexity of APOBEC expression in bats has recently been revealed (Hayward et al., 2018). Within the pteropid family, eighteen A3 proteins were identified, of which twelve are single deaminase A3Z1, one is a single deaminase A3Z2, four are composed of a single deaminase of a novel paralog termed ‘A3Z2B’, and one is a double deaminase of A3Z2B-Z3. Active expression of 13 of these proteins was identified in spleen tissue extracted from a black flying fox. The large expansion of Z1 is particularly unusual as this paralog has been independently lost in the mammalian clades Rodentia, Felidae, and Suidae (domestic and wild pigs) (LaRue et al., 2008) (Fig. 2b).

The A3 locus and its evolution in humans compared to other mammals is thoroughly described in Münk et al. (Munk et al., 2012) (Fig. 2c). A3A-G form the seven A3 proteins in the human A3 locus, and the dual-deaminase proteins form a complicated interrelationship of the A3Z1, A3Z2, and A3Z3 paralogs. The current organization likely arose through as many as 6 gene duplication events. In all known species, there is no evidence for duplication of the A3Z3 gene.

**APOBEC protein evolution.** The long-term evolution of the APOBEC proteins is of particular interest as it may indicate which of the proteins perform essential activities for host survival (and thus show low rates of evolution, referred to as purifying or negative selection) and which may be involved in host-pathogen interactions (and thus show rapid fixation of nonsynonymous mutations, referred to as positive or directional selection). Current evidence for the overall evolutionary trajectory is summarized in Table 1. When evaluating the evolution of AID using representatives from seven mammalian clades, the protein was shown to be under strict purifying evolution (Munk et al., 2012). Repeating the analysis for A3Z1, A3Z2, and A3Z2 demonstrated multimodal evolution and sliding-window analysis indicated that certain regions of the gene are under purifying selection and others are under positive selection. This multimodal relationship for A1 was also present but to a lesser degree than the A3 paralogs. It is likely that the regions under purifying selection are necessary for the direct function of the protein but the regions under positive selection allow for diversity in targets or are the targets of antagonizing viral proteins. Similar analysis with a more limited subset of clades indicated that A3Z1, A3Z2, and A3Z3 all showed dN/dS ratios greater than 1, consistent with overall positive selection (LaRue et al., 2008). When comparing whole genes between humans and chimpanzees, AID and A3A were under purifying selection, A3B, A3D, and A3G were under positive selection, and A3F was under positive selection in a sliding window analysis only (Sawyer et al., 2004). Comparing humans to a broader range of primates, A2 was under purifying selection and A3C was under positive selection in a sliding window analysis. A1 was under purifying selection in whole gene comparisons and positive selection under sliding window, consistent with the multimodal results seen above. Curiously, the sites under positive selection for A3G were not isolated to the regions targeted by the HIV-1 Vif protein as one may expect if the core function of positive selection was to limit retroviral antagonism escape. Altogether, the evidence implicates the A3 proteins and potentially some functionality of A1 as being involved in the pathogens arms race and being suitable targets for further investigation in an RNA virus context.

**Table 1:** Overview of APOBEC family proteins.

Name	Taxonomic Emergence	Evolutionary Pressure	Deaminase Domains (active)	Cellular Localization^a^	Preferred target Sequence (principally defined in ssDNA)
A1	Chordata	Multimodal (all mammals), purifying (whole gene), positive (sliding window)	1	n/C	5′-AC-3′
AID	Deuterostomes	Purifying	1	n/C	5′-WRC-3′
A2	Bony vertebrates (Euteleostomi)	Positive	?	N/C	?
A4	LECA?	?	?	?	?
A3Z1	Placental Mammals	Multimodal	1	?	?
A3Z2	Placental Mammals	Multimodal	1	?	?
A3Z3	Placental Mammals	Multimodal	1	?	?
A3A	Primates	Purifying	1 (N-A3Z1-C)	N/C	5′-TC-3′
A3B	Primates	Positive	2 (N-A3Z2-A3Z1-C)	N	5′-TC-3′
A3C	Primates	Positive (sliding window)	1 (N-A3Z2-C)	N/C	5′-TC-3′
A3D	Primates	Positive	2 (N-A3Z2-A3Z2-C)	C	5′-TC-3′
A3F	Primates	Positive (sliding window)	2 (N-A3Z2-A3Z2-C)	C	5′-TC-3′
A3G	Primates	Positive	2 (N-A3Z2-A3Z1-C)	C	5′-CC-3′
A3H	Primates	Unclear	1 (N-A3Z3-C)	N/C	5′-TC-3′

a Adapted from Salter et al. (2016). C, predominantly cytoplasmic; n/C, can traffic to nucleus but predominantly cytoplasmic; N/C, even localization; N, predominantly nuclear.

### 4.3 Bioinformatic evidence for RNA editing in SARS-CoV-2 and other coronavirus genomes

**C→U Mutational Asymmetry in SARS-CoV-2.** *Coronaviridae* are a family of single-stranded, positive-sense RNA viruses, of which there are seven known to infect and transmit in human populations (Table 2). HCoV-NL63 and HCoV-229E are seasonal alphacoronaviruses and HCoV-HKU1 and HCoV-OC43 are seasonal betacoronaviruses that all cause cold-like symptoms and circulate during the winter months (Gaunt et al., 2010). MERS-CoV, SARS-CoV-1, and SARS-CoV-2 are recently emergent betacoronaviruses that are responsible for large outbreaks of viral disease associated with high levels of morbidity and mortality. Coronaviruses, in common with other members of the *Nidovirales* order, encode a 3′-5′ exonuclease that dramatically reduces the effective error rate of the viral RNA-dependent RNA polymerase (Eckerle et al., 2007, 2010; Smith et al., 2013). Consequently, coronaviruses demonstrate a low rate substitution rate and minor differences in sequence diversity despite a genome size of nearly 30,000 bases.

**Table 2:** Characteristics of human-infective coronaviruses.

	Virus	Classification	Year of Discovery or Emergence
*Alphacoronavirus*	229E	Seasonal	1965 (Hamre and Procknow, 1966)
	NL63	Seasonal	2004 (van der Hoek et al., 2004)
*Betacoronavirus*	HKU1	Seasonal	2005 (Woo et al., 2005)
	OC43	Seasonal	1967 (McIntosh et al., 1967)
	MERS-CoV	Emergent	2012 (Zaki et al., 2012)
	SARS-CoV-1	Emergent	2003 (Ksiazek et al., 2003)
	SARS-CoV-2	Emergent	2019 (Wu et al., 2020; Zhou et al., 2020; Zhu et al., 2020)

Potential editing effects of APOBECs on RNA virus genomes is facilitated by the availability of an unprecedentedly large, expertly assembled and curated dataset of SARS-CoV-2 complete genome sequences that has accumulated since the start of the COVID-19 pandemic. The accuracy of the sequences obtained by a range of NGS technologies, combined with minimal evolutionary diversification in the few months since the start of its global spread provides a unique opportunity to investigate mutational events at very high resolution. All analyses of SARS-CoV-2 genomic sequence data report a preponderance of C→U transitions in the viral genome. Our early analysis (Simmonds, 2020b) of 1001 SARS-CoV-2 sequences indicated that 38–42% of all mutations in the queried datasets were due to C→U changes and that the ratio of C→U to U→C transitions was nearly six times higher than expected. Demonstrating that this was not an artifact of the sequencing or bioinformatics methods used, a parallel analysis of datasets of Ebola virus from historical strains and the 2014-16 West African outbreak demonstrated no such mutational asymmetry. Analysis of single nucleotide variations (SNVs) in a small collection of 34 SARS-CoV-2 sequences using the Wuhan-Hu-1 strain (MN908947) as a reference genome revealed 85 SNVs (Matyášek and Kovařík, 2020). Within these 85 SNVs, C→U changes showed an observed/expected ratio of 5.19 while U→C had a markedly lower ratio of 1.32. In Kilmczak et al. (Klimczak et al., 2020), a larger analysis of 32,115 isolates deposited on GenBank by late June tabled 251,273 mutations when compared to the reference MN908947 genome. Once duplicate mutations were removed and 12,156 mutational event identified, C→U and A→G mutations were shown to be the most prevalent single base substitutions. However, the removal of duplicate mutations may have biased the analysis against detection of independently occurring (homoplastic) C→U transitions at context-favored sites, as documented extensively in SARS-CoV-2 mutational analyses (Simmonds, 2020b). Additionally, a 3.5-fold asymmetry in C→U/U→C mutations was noted in samples from infected minks that had arisen during its transmission within mink farms in the Netherlands (Oude Munnink et al., 2021).

Most studies of mutation frequencies have been based upon the characterization of substitutions away from the SARS-CoV-2 global consensus sequence in individual genome sequences. Di Giorgio et al. (Di Giorgio et al., 2020) have contrastingly investigated the accumulation of mutations in the viral transcriptome via processing of RNA sequencing data from bronchoalveolar lavage fluid (BALF) samples of COVID-19 patients. Through this method, mutations occurring before fitness selection can be observed and potentially provide a better snapshot of the mutational spectrum of SARS-CoV-2 genomic sequences; for example, mutations that were likely to be lethal were readily identified. The authors state that the most common mutations in their datasets, namely A→G and T→C, were likely due to the action of adenosine deaminases that act on RNA (ADARs) while the proportion of C→U and G→A mutations was greater than that typically seen in the human transcriptome, suggesting an additional mechanism leading to the diversity.

The locations of C→U transition were loosely associated with the base and structure context of RNA deamination favored by APOBEC3 proteins. Base context in our analysis and Di Giorgio et al. indicated high preference for C→U deamination in the presence of upstream and downstream A or U. Klimczak et al. analyzed preference in trinucleotide motifs and found strong preference for uCn with total ablation of the phenotype in the context of an upstream C. Intriguingly, this extended context compared to the standard preference of several APOBECs for uC was similar to the A3A-driven editing of cellular mRNA sequences (Sharma et al., 2015) and in A3A-mediated mutations in ssDNA templates (Chan et al., 2015).

The other factor appearing to influence site preference in RNA sequences is the context of the target C in surrounding RNA secondary structure elements. Over half of the sites in human mRNA that were edited by A3A were flanked by short palindromic sequences and typically located in the unpaired termini of stem-loops (Sharma et al., 2016). Supporting this, an analysis of RNA structural contexts of edited sites in SARS-CoV-2 and rubella identified preferential C→U mutations in terminal loop compared to stem sequences (Klimczak et al., 2020), or in predicted paired compared to unpaired regions (Simmonds, 2020a).

### 4.4 Functional evidence for a role for APOBEC in editing and antiviral restriction of coronaviruses

The observed excess of C→U transitions, the nature of the 5′ and 3’ contexts where such transitions occur and the previously documented ability of several APOBEC paralogs to edit RNA templates make a compelling case for a role of APOBEC in editing of coronavirus genomes. However, functional studies have not, to date, unequivocally demonstrated the introduction of C→U transitions in viral RNA templates by APOBEC. In the most relevant functional study to date, interactions between human A3 proteins and HCoV-NL63 were demonstrated but without clear evidence of systematically biased RNA editing (Milewska et al., 2018). Infection of human airway epithelium culture upregulated A3A, A3C, A3D, and A3F. Transfection of LLC-Mk2 cells (rhesus macaque kidney) revealed deaminase-dependent virus restriction by A3C, A3F, and A3H but not A3A, A3E, or A3G. C→U and G→A point mutations were identified in virus serially passaged in A3A, A3C, A3E, A3F, A3G, and A3H overexpressing cells but did not result in hypermutation of progeny virus. However, potentially recapitulating interactions of APOBEC during assembly and consequent incorporation into retroviral particles, A3C, A3F, and A3H co-localized with the coronavirus nucleocapsid (N) protein through a catalytically-independent mechanism, while A3A, A3D, and A3G did not co-localize, associated with their differences in virus restriction.

Protein-protein interactions between A3 proteins and coronaviruses have furthermore been previously demonstrated in chimeric HIV-1 expressing SARS-CoV or HCoV-229E N (Wang and Wang, 2009). In these experiments, A3G packaged in the chimeric virions and was dependent on the carboxy-terminal self-association domain of the coronavirus N protein and not through binding to genomic RNA. Viral-like particles composed of SARS-CoV N and M proteins also co-packaged A3G.

A role of APOBEC in coronavirus restriction is further although indirectly supported by observations of the upregulation of A4, A3A, A3G, and A3H and downregulation of A3C in 10.13039/100013479BALF samples from patients hospitalized with COVID-19 although there were no significant changes in APOBEC expression in peripheral blood mononuclear cells (PBMCs) (Xiong et al., 2020). Analysis of immune cells in BALF samples from nine patients demonstrated upregulation of A3A and A3B in macrophages and A3G in NK cells (Liao et al., 2020). Tissue expression in two post-mortem lung biopsies demonstrated upregulation of A3A only (Blanco-Melo et al., 2020). While these findings have been advanced as supporting evidence for a role of APOBECs in controlling infections, it should be noted that their expression, as ISGs, is primarily dependent on IFN-induced signaling, a process that activates hundreds of cellular genes that play roles in innate immunity, irrespective of pathway. Demonstration of specific interactions of individual APOBEC proteins with coronaviruses and functional effects of their expression on virus replication is required to substantiate these associations.

**Evolutionary consequences of C**→**U mutational bias in coronaviruses.** Irrespective of the underlying mutational mechanisms, we have reviewed the substantial evidence for a potent editing process that drives C→U mutations into the genomes of coronaviruses and potentially other RNA viruses in certain favored sequence and RNA structural contexts. In evolutionary terms, this driver of virus sequence change is quite distinct from the occurrence of random mutations through misincorporation errors during transcription that is assumed in standard evolutionary models. Apart from the restriction of mutations to C→U changes (or G→A if the negative-sense strand is edited), sequence changes are also primarily distributed in mutational hotspots that may lead to the appearance of convergent changes between otherwise genetically unlinked strains. For example, several C→U mutations occurred independently among variants of SARS-CoV-2 that were distributed into different parts of the tree (Simmonds, 2020b), and therefore conflicted with relationships reconstructed from phylogenetically informative sites. A high frequency of homoplastic mutations were similarly described in a larger analysis of 7666 genome sequences of SARS-CoV-2 (van Dorp et al., 2020). Conflicts between the distribution of C→U changes with underlying phylogenetic relationships hampers evolutionary reconstructions and rate calculations (Mavian et al., 2020).

The occurrence of C→U hypermutation in SARS-CoV-2 also has a substantial skewing effect on relative frequencies of non-synonymous (dN - amino acid changing) to synonymous substitutions (dS) occurring during virus evolution. Most viral sequence diversity is generated through fixation of neutral substitution that do not influence the fitness of the virus. These typically are synonymous, and comparative analyses of virus sequence datasets typically reveals low dN/dS ratios indicative of purifying selection, such as the 0.1 ratio found in a dataset of Ebolavirus genome sequences during its recent epidemic spread in Central and West Africa (Simmonds, 2020b). Contrastingly, the high dN/dS ratio between strains (Simmonds, 2020b; van Dorp et al., 2020) or between SARS-CoV-2 and the most closely related bat sarbecovirus, RaTG13 (Wang et al., 2020) indicates that much of the sequence diversity in SARS-CoV-2 is driven by mutational mechanisms distinct from a process of random mutation and fixation assumed in models of neutral evolution.

Furthermore, a substantial proportion of amino acid changes that evolve within SARS-CoV-2 populations originate from non-synonymous changes induced by C→U transitions (Matyášek and Kovařík, 2020; Simmonds, 2020b), rather than necessarily representing evolutionarily selected phenotypic changes that might be expected to occur as the virus adapts to replication and transmission in a new host (MacLean et al., 2020). The occurrence of C→U driven amino acid changes with their likely often mildly deleterious phenotypes and likelihood of reversion considerably complicates analyses of the critical protein changes associated with adaptation to a human host. The occurrence of large numbers of homoplastic sites similarly complicates analyses of sites under positive selection and recombination analysis. Finally, the sheer number of excess C→U changes observed in SARS-CoV-2 genomes (>40% of observed substitutions in some datasets) has a distorting effect on estimates of SARS-CoV-2 substitution rates, particularly as many sites may rapidly revert over time (van Dorp et al., 2020).

The long-term effects of the (C→U)/(U→C) transition asymmetry on virus diversification are difficult to predict. However, the compositional abnormalities of other coronaviruses, particularly human seasonal coronaviruses, might plausibly be attributed to the long term outcome of prolonged C→U editing (Berkhout and van Hemert, 2015; Simmonds, 2020b; Woo et al., 2007). For example, the enrichment of U (40%), depletion of C (13%) and intermediate frequencies of A and G bases (28% and 19%) in HCoV-HKU1 has been proposed to have been driven by APOBEC associated mutations (Woo et al., 2007). Of potential relevance to exploring host differences in APOBEC activity in different hosts, there is no compositional asymmetry and a relatively higher G + C contents in bat sarbecoviruses from which SARS-CoV-2 derives. The intensity of C→U mutational pressure observed in human SARS-CoV-2 sequences since its zoonotic emergence may therefore reflect a less permissive, hostile internal cellular environment in human cells than might be found in a better co-adapted, virus-tolerized immune system of the average bat (Baker et al., 2013).

Downstream induction of inflammatory cytokine and adaptive immune system responses may contribute the exaggerated and damaging inflammatory pathology observed in COVID-19 cases. The pronounced activity of APOBEC in human cells may derive from an inability of a bat-adapted virus to counteract the human APOBEC editing pathway. The varying degrees of base asymmetry and G + C contents of human seasonal coronaviruses may then be indicative of the varying times they have circulated in humans following their acquisition from bats (Corman et al., 2018). SARS-CoV-2 may follow their evolutionary trajectory if infections become permanently established in human populations.

## 5 A perspective on future functional investigations of APOBEC-mediated editing of SARS-CoV-2 and other RNA viruses

APOBECs have been shown to be important for host and retroviral function, and the possibility that they play a role in RNA viral evolution would substantially expand the scope of their antiviral activities. However, part of the difficulty in studying the process functionally is the plethora of different APOBECs in human with likely different virus, RNA, and DNA targeting and biological activities. Furthermore, the number and identities of APOBECs in high variable in mammals; for example, the mouse which might otherwise represent a suitable *in vivo* model for APOBEC function in whole host defense possesses only one A3 gene compared to seven or more in primates and bats.

Functional studies using human cell lines are similarly hampered by the current lack of information of which APOBEC protein might mediate the observed RNA editing in coronaviruses and in other RNA viruses, or whether their might exist currently uncharacterized defense pathways. Among the members of the diverse family, A1 and A3, with their evidence for extensive positive selection during mammalian evolution, represent the frontrunners for deaminase-dependent mutation of RNA viruses. A1 is an unlikely candidate for SARS-CoV-2 editing in humans, as its target specificity and restricted hepatic expression of the protein limits its ability to mutate what is principally a respiratory pathogen. Among the A3 proteins, it may be possible to exclude A3G from contributing to SARS-CoV-2 deamination as its upstream base preference for C preference for G does not match the observed contexts of editing sites in SARS-CoV-2 at. Beyond that, one may consider intracellular distributions of this proteins as limiting factors, as SARS-CoV-2 and other coronaviruses spend their entire intracellular life cycles in the host cytoplasm, but first the intracellular distributions of the A3 proteins in different activation contexts should be better defined. Further, it is conceivable that, similar to HIV-1, any editing of SARS-CoV-2 RNA may be enhanced by packaging of A3 proteins in the viral capsid with the bulk of deleterious editing taking place in the subsequent cellular infection. In this way, experiments aimed at identifying protein-protein interactions between the viral capsid and host proteins may assist in narrowing the breadth of proteins that should be investigated. In all cases, *trans* complementation of the editing phenotype by gene transfection may be difficult to achieve *in vitro* if there are functionally required interacting cofactors or activated intracellular states. Further research into the protein family is warranted (Box 1).

Box 1 **Outstanding research questions**

A more thorough understanding of the general evolution and role of the APOBEC family proteins will benefit from addressing the following research themes:

• APOBEC emergence and evolution
  o Identification of the presence of A4 homologs in ray-finned fish or protostomes.
  o Evolutionary relationship between AID and A2.
  o Significance of the loss of A3Z1 in multiple branches of placental mammals.
  o Study of the activity of APOBECS in the Carnivora genus motivated by the activity of feline and potentially mink homologs.
• APOBEC function
  o Function of A2 or A4 *in* vitro and in *vivo.*
  o Necessary cofactors for deaminase activity in a variety of cell and activation contexts. Tissue and cellular distribution of these cofactors.
  o Functional importance of dual-deaminase domains in A3 proteins in placental mammals.
  o The role of base context and secondary structure motifs on APOBEC deamination activity on ssRNA.
  o Intracellular localization of APOBEC proteins at rest and in activated contexts.
• APOBEC-virus interactions
  o Dependence of deamination phenotype on protein-protein interactions with viral proteins.
  o Mechanisms of escape of APOBEC targeting for influenza and vaccinia virus.
  o Cellular location and virus replication stage where APOBEC-mediated RNA virus genome editing occurs
  o Packaging of APOBEC in non-retrovirus virions?

Alt-text: Box 1

Whether or not the C→U phenotype is driven by APOBEC or by currently uncharacterized intracellular mechanism does not mitigate the functional significance of the observed hypermutation phenotype. Homoplastic transitions in the viral genome impair attempts to construct phylogenetic trees used for molecular epidemiology investigations and may result in spurious associations between unrelated lineages. Were these mutations to be driven by a host element rather than misincorporation errors leading to selection, it would also undermiuneunder the assumptions of neutral evolution underpinning much of the conceptual basis of virus evolutionary models. Lastly, the overrepresentation of these transitions in nonsynonymous mutations underpins its role in generating protein diversity. The long term effect of these potentially driven mutations to viral fitness is uncertain but will contribute to the overall population diversity.

## Funding

The work was supported by a 10.13039/100004440Wellcome Investigator Award Grant (WT103767MA).

## CRediT authorship contribution statement

**Jeremy Ratcliff:** Conceptualization, Writing - original draft, preparation, Writing - review & editingWriting – review & editing. **Peter Simmonds:** Writing - original draft, Writing - Draft extension, Writing - review & editing, Funding acquisition.
